# PLK2 as a key regulator of glycolysis and immune dysregulation in polycystic ovary syndrome

**DOI:** 10.3389/fimmu.2025.1610713

**Published:** 2025-09-11

**Authors:** Hua Ma, Dahe Qi, Yating Xu, Tingting Shang, Yu Si, Wenyue Chen, Hongting Zhao, Qingling Ren

**Affiliations:** ^1^ Department of Gynecology, Affiliated Hospital of Nanjing University of Chinese Medicine, Nanjing, Jiangsu, China; ^2^ The Chinese Clinical Medicine Innovation Center of Obstetrics, Gynecology, and Reproduction in Jiangsu Province, Nanjing, Jiangsu, China; ^3^ Department of Neurology, Dongzhimen Hospital, Beijing University of Chinese Medicine, Beijing, China

**Keywords:** polycystic ovary syndrome, glycolysis, endothelial cells, PLK2, single cell sequencing

## Abstract

**Background:**

Polycystic ovary syndrome (PCOS) is a highly heterogeneous endocrine-metabolic disorder. Ovarian stromal cells influence follicular development and ovulation by secreting cytokines. Glycolysis, a central pathway of glucose metabolism, plays a crucial role in the pathogenesis of PCOS. However, the precise mechanisms underlying dysregulated glycolysis in ovarian stromal cells in PCOS remain unclear.

**Methods:**

Seurat and CellChat were employed to analyze single-cell RNA sequencing (scRNA-seq) data, incorporating glycolysis scoring and cell-cell communication analysis. Three independent bulk RNA-seq datasets were integrated to identify key genes. Immune infiltration was assessed using CIBERSORT, ESTIMATE, and ssGSEA algorithms. Functional enrichment analysis (GO, KEGG, and Hallmark) was performed to annotate PLK2-related pathways. Finally, a dehydroepiandrosterone (DHEA)-induced PCOS rat model was constructed to validate the critical role of PLK2 expression in PCOS.

**Results:**

Single-cell sequencing analysis revealed that endothelial cells in the ovarian stroma of PCOS exhibited the highest glycolytic activity and increased intercellular communication, particularly interacting with fibroblasts via the PPIA-BSG ligand-receptor pair. Integrated transcriptomic analysis identified PLK2 as a central regulatory gene of endothelial glycolysis (AUC > 0.85). Functional enrichment analysis further demonstrated that high PLK2 expression was closely associated with extracellular matrix (ECM) remodeling and promoted chronic inflammation and ovarian fibrosis by activating the NF-κB and IL-17 signaling pathways. Immune infiltration analysis indicated that PCOS patients with high PLK2 expression exhibited enhanced pro-inflammatory responses, increased neutrophil recruitment, and impaired T-cell function, suggesting a shift toward an inflammatory ovarian microenvironment. The DHEA-induced PCOS rat model further confirmed the critical role of PLK2 in disease progression and glycolytic dysregulation.

**Conclusion:**

This study establishes PLK2 as a key regulator of glycolysis and immune imbalance in PCOS, highlighting its pivotal role in the metabolic-immune crosstalk within the ovarian microenvironment. These findings suggest that targeting PLK2 may be a potential therapeutic strategy for alleviating glycolytic dysregulation and chronic inflammation in PCOS.

## 1 Introduction

Polycystic ovary syndrome (PCOS) is a highly heterogeneous gynecological endocrine-metabolic disorder. Its core diagnostic criteria include ovulatory dysfunction, hyperandrogenism (clinical or biochemical), and polycystic ovarian morphology (1, 2). Due to the lack of a single biomarker or imaging standard for independent diagnosis, the current diagnostic practice relies on the revised Rotterdam criteria, which assess symptom combinations (3). The pathogenesis of PCOS involves a complex interplay of genetic predisposition, hormonal imbalance, metabolic dysregulation, and environmental factors (4).

Ovarian stroma is a critical component of ovarian tissue, encompassing all cellular components except follicles, including endothelial and immune cells (5, 6). Historically, research has predominantly focused on the interactions between granulosa cells and oocytes within follicles. In contrast, the role of ovarian stromal cells in follicular development and ovulation has gained increasing attention. Beyond providing structural support, stromal cells actively regulate follicular maturation and ovulation by secreting various growth factors, cytokines, and hormones (7, 8). In PCOS, abnormal stromal cell function may contribute to increased stromal area, impaired vascularization, and chronic inflammation, ultimately disrupting follicular development and ovulatory capacity (9).

Glycolysis, the core pathway of glucose metabolism, plays a critical role in developing PCOS. Metabolomic studies have indicated that ovarian glucose metabolism in PCOS is characterized by decreased glucose uptake and consumption in oocytes, enhanced glycolytic flux in follicular fluid, and suppressed glucose metabolism in granulosa cells (10–12). Zhao et al. (13) utilized NMR and GC-MS to analyze plasma metabolites and observed enhanced glycolysis alongside inhibition of the tricarboxylic acid (TCA) cycle in PCOS patients. Aberrant glycolytic activity in ovarian oocytes, granulosa cells, and systemic metabolic tissues is closely linked to PCOS pathogenesis (14). Granulosa cells convert glucose into lactate and pyruvate, supplying energy to oocytes (15). Notably, PCOS follicles require increased lactate to sustain growth (16); however, experimental studies have shown that key rate-limiting glycolytic enzymes, such as PKM2 and LDHA, are downregulated in PCOS rat ovaries, leading to reduced lactate production (17). This imbalance between increased energy demand and insufficient supply contributes to follicular developmental arrest in PCOS.

Nevertheless, the precise mechanisms underlying glycolytic dysregulation in ovarian stroma remain unclear. The cell-type-specific reprogramming of glycolysis and its impact on systemic metabolic dysfunction in PCOS remains largely unexplored. To address these knowledge gaps, omics technologies, particularly single-cell RNA sequencing (scRNA-seq) and bulk transcriptomics have emerged as powerful tools for deciphering cell-specific molecular characteristics and systemic metabolic interactions in complex diseases. scRNA-seq enables the identification of cellular heterogeneity and characterization of dynamic cellular changes and intercellular interactions at single-cell resolution (18). By revealing transcriptomic signatures of different ovarian cell populations, this approach provides insights into the distinct roles of various cell types in follicular development and steroidogenesis (19). Complementary to scRNA-seq, bulk transcriptomics offers a comprehensive view of gene expression patterns at the tissue level, facilitating the identification of dysregulated pathways and potential therapeutic targets. Integrating single-cell resolution data with bulk transcriptomic breadth provides a robust framework for understanding PCOS complexity (20), from cellular heterogeneity to systemic metabolic dysregulation, ultimately advancing disease mechanism research and therapeutic innovation.

In this study, we leveraged the synergistic strengths of scRNA-seq and bulk transcriptomics to systematically investigate glycolytic dysregulation in PCOS, identifying PLK2 as a key regulatory gene in endothelial cell glycolysis and cell-cell communication. This approach offers novel insights into the regulatory mechanisms linking glycolysis and PCOS pathogenesis.

## 2 Materials and methods

### 2.1 Data sources

The scRNA-seq dataset GSE268919 (18) was obtained from the Gene Expression Omnibus (GEO) database (https://www.ncbi.nlm.nih.gov/gds). This dataset includes six ovarian tissue samples, comprising three from PCOS mice and three from control mice. Notably, these samples were derived from whole ovarian tissue, allowing for a comprehensive representation of the ovarian microenvironment.

To further investigate transcriptomic characteristics associated with PCOS, three additional datasets were retrieved from the GEO database: GSE34526 (21), GSE95728, and GSE106724 (22). The GSE34526 dataset, based on the GPL570 platform, includes granulosa cell samples from seven PCOS patients and three healthy controls. The GSE95728 dataset, based on the GPL16956 platform, consists of granulosa cell samples from seven PCOS patients and seven controls. Additionally, the GSE106724 dataset, based on the GPL21096 platform, comprises granulosa cell samples from eight PCOS patients and four controls. We used these granulosa cell datasets as representatives to investigate the transcriptomic alterations in the ovaries of PCOS patients.

### 2.2 Single-cell RNA sequencing analysis

scRNA-seq data were processed using the Seurat R package (version 4.3.1) (23). Low-quality cells were filtered out based on the following criteria: fewer than 200 detected genes, nFeature_RNA>5000, nCount_RNA>3000, and mitochondrial gene percentage>10%. After data normalization and identification of highly variable genes, dimensionality reduction was performed using principal component analysis (PCA). Cell clustering was conducted using the FindNeighbors and FindClusters functions with a resolution of 0.5, and visualization was achieved via Uniform Manifold Approximation and Projection (UMAP). Cluster-specific marker genes were identified using the FindAllMarkers function. Finally, cell clusters were annotated based on reference information from the CellMarker database (http://bio-bigdata.hrbmu.edu.cn/CellMarker/).

### 2.3 Glycolysis scoring and cell-cell communication analysis

To evaluate glycolytic activity at the single-cell level, a list of glycolysis-related genes from the Kyoto Encyclopedia of Genes and Genomes (KEGG) pathway (hsa00010) was compiled, including key enzymes and regulators such as LDHA, PKM2, PFKP, and ALDOA. Glycolysis scores were calculated using the AddModuleScore and PercentageFeatureSet functions in Seurat and single-sample gene set enrichment analysis (ssGSEA) from the Gene Set Variation Analysis (GSVA) package. Higher glycolysis scores indicated increased glycolytic activity. Visualization of glycolysis activity across different cell types was performed using violin plots and UMAP feature plots.

### 2.4 Cell communication analysis

Cell communication analysis was performed using the CellChat R package (version 1.6.1). The CellChatDB database was utilized to infer potential intercellular interactions involving endothelial cells and other cell types, highlighting the overexpressed ligand-receptor pairs.

### 2.5 RNA sequencing analysis and identification of differentially expressed genes

The GSE34526, GSE95728 and GSE106724 datasets were merged and normalized using the “sva” package, and the expression values were corrected in batches to remove batch effects (24). PCA analysis was performed after removing the batch effects. Differentially expressed genes (DEGs) between PCOS and control groups were identified using the LIMMA package (25). Volcano plots were generated to highlight DEGs. Adjusted p-values were considered to account for false positives, and DEGs were defined by an adjusted *P* < 0.05 and |log2FC| >0.5.

### 2.6 Enrichment analysis

Gene Ontology (GO) and KEGG pathway enrichment analyses of DEGs were conducted using the “clusterProfiler” and “ggplot2” R packages. Additionally, hallmark gene set enrichment analysis (GSEA) was performed to explore the cellular localization, biological processes, and signaling pathways associated with these genes in PCOS.

### 2.7 Assessment of immune-related transcriptomic signatures

To evaluate immune-related transcriptional responses in ovarian granulosa cells, we applied the CIBERSORT algorithm to bulk RNA-seq data using the LM22 signature matrix to infer expression patterns associated with 21 immune cell types. This analysis does not represent actual immune cell abundance but rather indicates potential immune signal responsiveness reflected in granulosa cell transcriptomes.

In addition, single-sample Gene Set Enrichment Analysis (ssGSEA) was performed using immune-related gene sets from the MSigDB database to calculate enrichment scores of immune pathways for each sample. This enabled the comparison of immune signaling activity between samples with high and low PLK2 expression.

### 2.8 Animal model establishment and validation

Female Sprague-Dawley (SD) rats (3 weeks old) were purchased from VITAL RIVER Laboratory Animal Technology Co., Ltd. All animals were housed in a specific pathogen-free (SPF) facility under controlled conditions (22–24 °C, 65 ± 5% humidity, 12-hour light/dark cycle) for seven days to acclimate. The experimental protocol was reviewed and approved by the Institutional Animal Care and Use Committee of the Affiliated Hospital of Nanjing University of Chinese Medicine (approval code: 2024DW-095-01).

Twelve rats were randomly divided into two groups (n = 6 per group): a control group and the DHEA-induced PCOS model group. The PCOS model was induced by daily subcutaneous injection of DHEA (60 mg/kg·d) dissolved in 0.2 mL sesame oil for 21 consecutive days. The control group received an equivalent volume of sesame oil. From day 8 of DHEA administration until the end of the experiment, vaginal smears were collected daily between 9:00 and 10:00 AM to monitor estrous cycles.

After 21 days of DHEA treatment, rats were fasted overnight before undergoing an intraperitoneal glucose tolerance test (IPGTT) the following morning. Subsequently, the animals were euthanized, and blood and ovarian tissues were collected. Serum levels of luteinizing hormone (LH), follicle-stimulating hormone (FSH), anti-Müllerian hormone (AMH), and testosterone (T) were measured using ELISA kits (LAPUDA, China).

### 2.9 HUVEC culture and DHEA treatment

Human umbilical vein endothelial cells (HUVECs) were obtained from Haixing Biosciences (Suzhou, China) and cultured in endothelial cell medium (TCH-G406, Haixing Biosciences, Suzhou, China) at 37 °C in a humidified incubator containing 5% CO_2_. To mimic the hyperandrogenic microenvironment of PCOS, cells were treated with dehydroepiandrosterone (DHEA) (D106380, Aladdin Biotech, Shanghai, China) at gradient concentrations (0-140 μM) for 24 h. The effects on cell viability and glycolysis-related gene expression were subsequently evaluated.

### 2.10 Cell viability assay (CCK-8)

Cell viability after 24 h of DHEA treatment at different concentrations was measured using a CCK-8 assay kit (C0005, Targetmol, USA). Briefly, 10 μL of CCK-8 working solution was added to each well, incubated at 37°C for 2 h, and the absorbance was recorded at 450 nm using a microplate reader.

### 2.11 SiRNA transfection and experimental grouping

Small interfering RNA (siRNA) targeting PLK2 was designed and synthesized by GenePharma (Shanghai, China), and primer sequences are provided in **Supplementary Table 1**. Transfection was performed using Lipofectamine 2000 (40802ES03, Yeasen, Shanghai, China) according to the manufacturer’s instructions. Knockdown efficiency was verified by quantitative PCR. Four experimental groups were established: NC (untreated control), NC + DHEA (20 μM DHEA), siPLK2, and siPLK2 + DHEA (20 μM DHEA). After transfection, cells were treated for an additional 24 h before being harvested for qPCR analysis.

### 2.12 Quantitative real-time RT-PCR

Total RNA was isolated from cells using the FastPure Cell/Tissue Total RNA Isolation Kit (RC101-01, Vazyme, Nanjing, China). The extracted RNA was then reverse transcribed into complementary DNA (cDNA) using SuperMix (R323-01, Vazyme, Nanjing, China). Quantitative polymerase chain reaction (qPCR) was performed using the QuantStudio 5 system and SYBR Green PCR Master Mix (Q311-02, Vazyme, Nanjing, China). For each sample, qPCR was conducted in triplicate. The primers used for qPCR analysis are listed in **Supplementary Table 2**.

### 2.13 Western blot

Ovarian tissues from rats were lysed in RIPA buffer supplemented with protease inhibitors. Total protein concentrations were determined using a BCA protein assay kit. Equal amounts of protein were separated by SDS-PAGE and transferred onto PVDF membranes. Membranes were blocked with 5% BSA and incubated overnight at 4°C with primary antibodies against PLK2 (1:500, Affinity, DF4470, China) and Actin (1:10,000, Proteintech, 66009-1-Ig, Wuhan, China). After incubation with HRP-conjugated secondary antibodies (Proteintech, SA00001-2, Wuhan, China), protein bands were visualized using ECL reagents and quantified using ImageJ software.

### 2.14 Immunohistochemistry

IHC staining was performed to assess PLK2 expression in ovarian tissues. Paraffin-embedded ovarian sections were deparaffinized, rehydrated, and subjected to antigen retrieval using citrate buffer. After blocking, sections were incubated overnight at 4°C with a primary antibody against PLK2 (1:900 dilution; Affinity DF4470, China), followed by HRP-conjugated secondary antibody incubation at 37°C for 30 minutes. Staining was visualized using a slide scanner.

### 2.15 Statistical analysis

Bioinformatics analyses were conducted using R (version 4.3.1), while statistical analysis and visualization were performed using GraphPad Prism 9.0. Data are presented as mean ± SEM from three independent experiments. Statistical comparisons were conducted using Student’s t-test, with significance thresholds set at *P* < 0.05 (*P < 0.05, **P < 0.01, ***P < 0.001).

## 3 Results

### 3.1 Single-cell profiling identifies glycolysis-active endothelial cells as key players in PCOS pathogenesis

**Figure 1:**
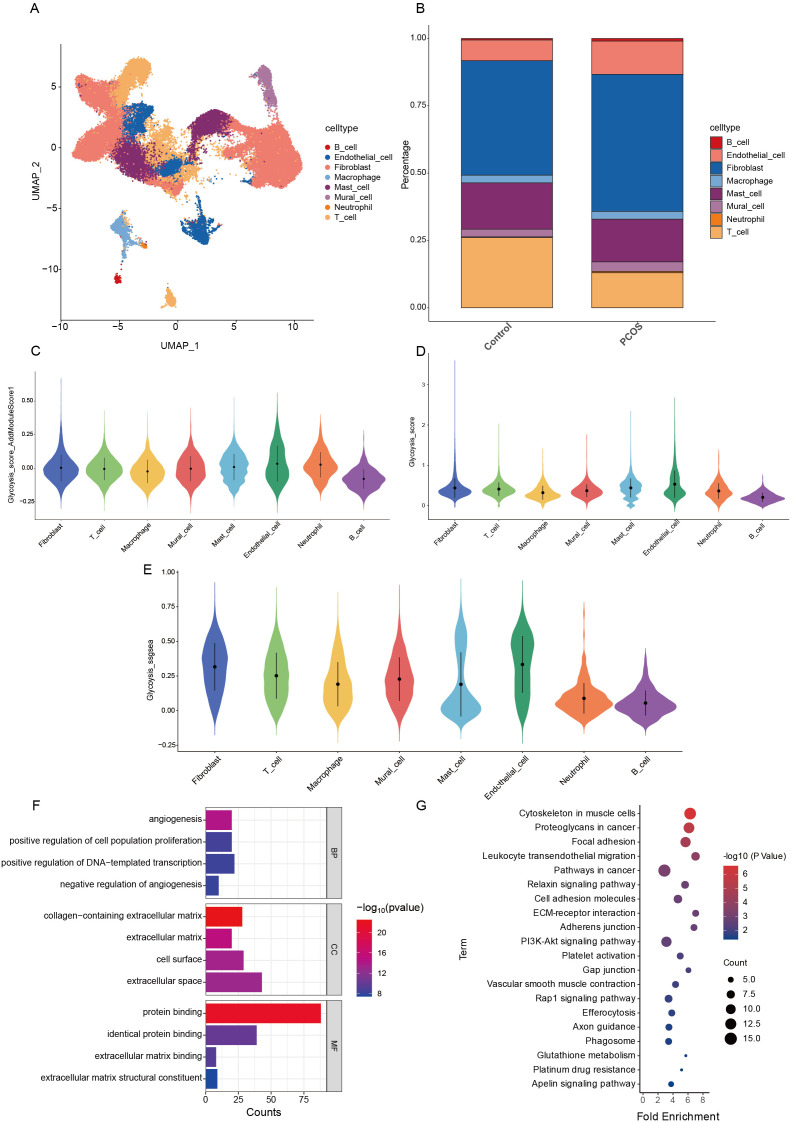
Single-cell RNA sequencing analysis and glycolysis scoring in PCOS. **(A)** UMAP visualization of single-cell RNA sequencing (scRNA-seq) data. **(B)** Distribution of different cell types in the ovary. **(C)** AddModuleScore algorithm to assess glycolysis scores of different cell types in PCOS. **(D)** Percentage Feature Set algorithm to assess glycolysis scores of different cell types in PCOS. **(E)** The ssgsea algorithm to assess the glycolysis score of different cell types in PCOS. **(F)** GO enrichment analysis of DEGs in PCOS-associated endothelial cells. **(G)** KEGG enrichment analysis of DEGs in PCOS-associated endothelial cells.

Following normalization and rigorous quality control of single-cell transcriptomic data, we retained 55892 high-quality cells for analysis (**Supplementary Figures S1, S2**). With a clustering resolution parameter set at 0.5, we identified 21 distinct cell subpopulations (clusters 0–20), including endothelial cells, fibroblasts, macrophages, and T cells. UMAP visualization revealed significant compositional differences between PCOS and control groups, suggesting disease-associated cellular heterogeneity (**Figures 1A, B**).

To evaluate glycolytic activity across ovarian cell types, we employed AddModuleScore, PercentageFeatureSet, and ssGSEA algorithms. Endothelial cells exhibited the highest glycolysis scores relative to other cell types, identifying them as the most glycolysis-active cell population in PCOS ovaries (**Figures 1C–E**). DEGs associated with high glycolysis in PCOS endothelial cells were subjected to GO and KEGG pathway enrichment analyses. GO analysis revealed enrichment in biological processes such as angiogenesis, positive regulation of cell proliferation, and extracellular matrix (ECM) organization. Notably, genes associated with ECM binding and protein binding were also enriched, indicating a potential role in ovarian microenvironment remodeling in PCOS (**Figure 1F**). KEGG pathway analysis highlighted multiple glycolysis-related pathways, including the PI3K-Akt signaling pathway, ECM-receptor interactions, and leukocyte transendothelial migration. These pathways are critically linked to cellular adhesion, vascular smooth muscle contraction, and platelet activation, potentially contributing to metabolic dysregulation and immune infiltration in PCOS (**Figure 1G**).

### 3.2 Endothelial-centric crosstalk in the PCOS ovarian microenvironment

**Figure 2:**
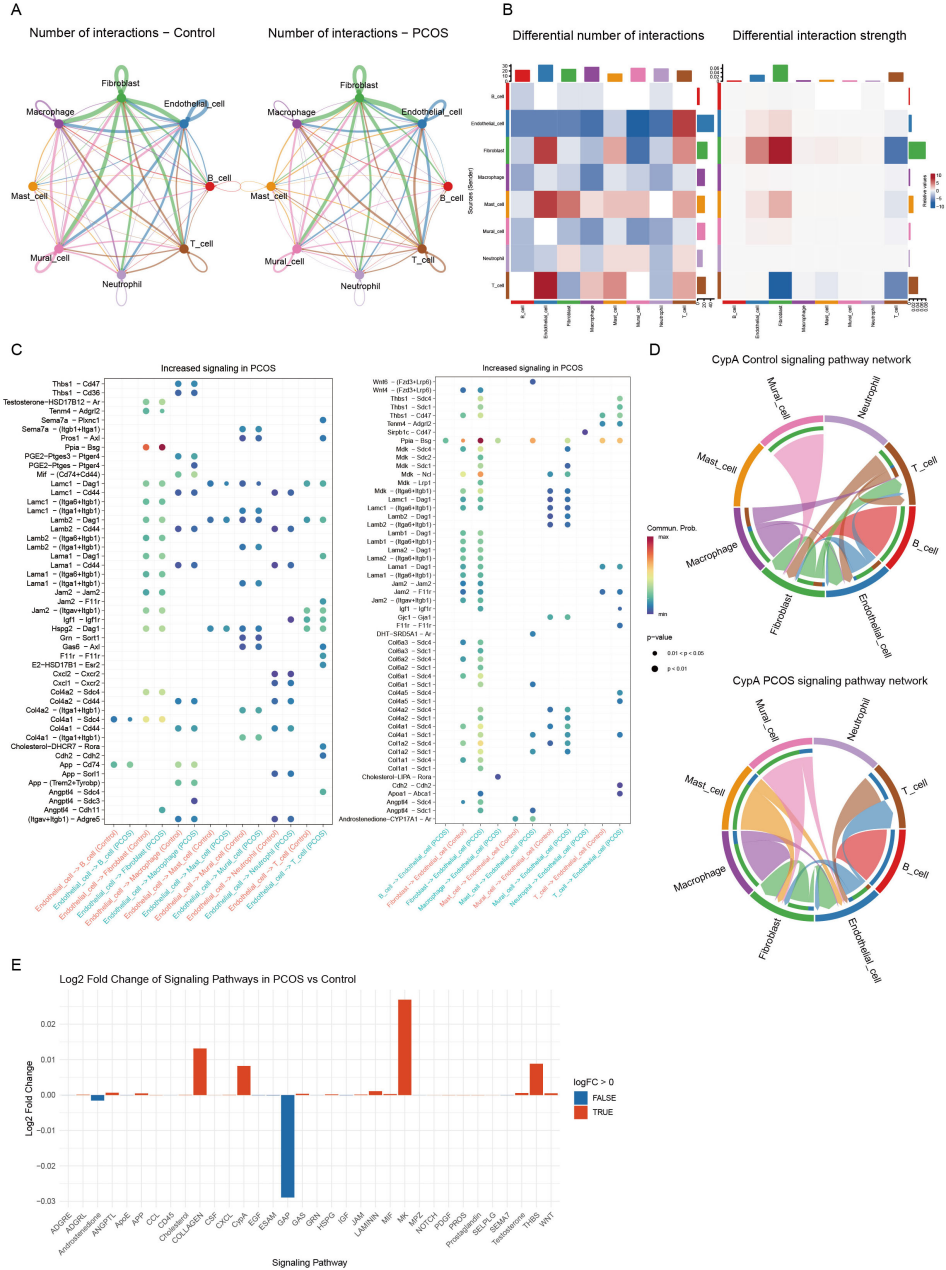
Analysis of intercellular communication networks. **(A)** Comparison of the number of intercellular interactions between the control group (left) and the PCOS group (right). The thickness of the lines represents the number of interactions. **(B)** Heatmaps illustrating differences in the number of interactions (left) and interaction strength (right) between the PCOS and control groups. Red indicates an increase in interaction frequency or strength, while blue represents a decrease. **(C)** Bubble plots showing signaling pathways with increased activity in PCOS, the left plot indicates the effect of endothelial cells on other cells, and the right plot demonstrates the effect of other cells on endothelial cells. Each dot represents a specific ligand-receptor pair, its size indicates the strength of the interaction, and the color reflects statistical significance. **(D)** Chord diagrams visualizing the CypA signaling pathway in the control group (top) and PCOS group (bottom), demonstrating the probability of intercellular communication between different cell types. **(E)** Bar plot displaying the log2 fold change in signaling pathway activity in the PCOS group compared to the control group. Red bars represent upregulated pathways, while blue bars indicate downregulated pathways.

To further investigate the interactions between different cell types in the context of glycolysis, we performed cell-cell communication analysis. Significant differences in intercellular interactions were observed between PCOS and control groups, particularly in endothelial cells. In PCOS, endothelial cells exhibited increased interactions with multiple cell types, including fibroblasts, T cells, and mast cells (**Figures 2A, B**).

Notably, the PPIA-BSG ligand-receptor pair played a pivotal role in endothelial-fibroblast interactions, forming an endothelial-centered signaling network. This network contributed to inflammatory responses and ECM remodeling, both of which are hallmarks of PCOS. Additionally, the interactions of Angptl4-Cdh5 and Angptl4-Sdc1 were significantly enriched in PCOS, underscoring the role of endothelial cells in angiogenesis and cell adhesion (**Supplementary Figure S3**, **Figure 2C**).

Chord diagram visualization further demonstrated enhanced endothelial-fibroblast interactions via the CypA signaling pathway in PCOS (**Figure 2D**). Comparative pathway analysis revealed a significant upregulation of CypA-mediated interactions in PCOS (**Figure 2E**). These findings suggest that glycolysis-driven metabolic reprogramming in endothelial cells may reshape the ovarian microenvironment by activating fibroblasts and disrupting immune homeostasis, ultimately exacerbating PCOS pathogenesis.

### 3.3 Integrated transcriptomic analysis identifies PLK2 as a core glycolysis-linked gene in PCOS

**Figure 3:**
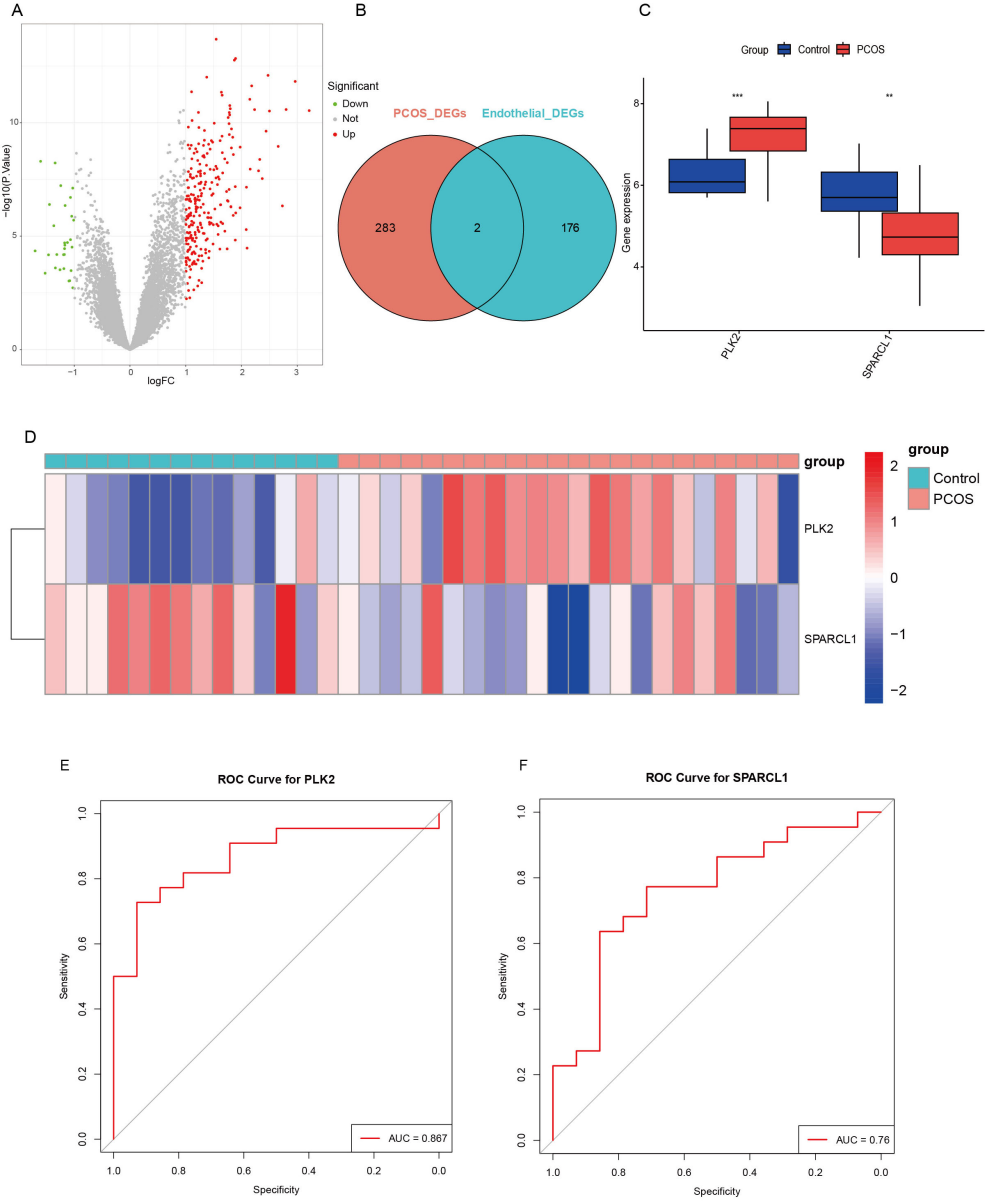
Integrated transcriptome analysis identifies PLK2 as a core glycolytic-related gene in PCOS. **(A)** Volcano plot showing DEGs between PCOS and control groups. Red dots indicate upregulated genes, green dots indicate downregulated genes, and gray dots indicate non-significant genes. **(B)** Venn diagram showing the overlap between DEGs in PCOS and endothelial cell-specific DEGs with high glycolytic activity. The intersection highlights shared DEGs. **(C)** Box plots comparing the expression levels of PLK2 and SPARCL1 between the PCOS and control groups. Statistical significance is indicated by ** (*P* < 0.01) and *** (*P* < 0.001). **(D)** Heatmap depicting the expression levels of PLK2 and SPARCL1 in samples from the PCOS and control groups. The color gradient represents the relative expression levels, with red indicating up-regulation and blue indicating down-regulation. **(E)** ROC curve of PLK2. **(F)** ROC curve of SPARCL1.

To systematically identify key drivers of endothelial dysfunction in PCOS, we integrated three human ovarian granulosa cell transcriptomic datasets (GSE34526, GSE95728, GSE106724), including 22 PCOS and 14 control samples, to identify conserved transcriptomic features associated with PCOS. After batch correction using the ComBat algorithm (**Supplementary Figure S4**), PCA confirmed the effective removal of technical variation (**Supplementary Figure S4**). Differential expression analysis (|log2FC| > 1, adjusted *P* < 0.05) identified 285 DEGs, including 256 upregulated and 29 downregulated genes (**Figure 3A**). Intersecting these DEGs with glycolysis-related endothelial genes identified from single-cell analysis revealed two core overlapping genes: PLK2 and SPARCL1 (**Figures 3B–D**). Receiver operating characteristic (ROC) curve analysis demonstrated superior diagnostic performance of PLK2 (AUC=0.867) over SPARCL1 (AUC=0.76) in distinguishing PCOS from control samples (**Figures 3E, F**).

### 3.4 Validation of PLK2 expression in human ovarian single-cell dataset

To validate the expression profile of PLK2 in human ovarian cells, we analyzed the ovarian single-cell transcriptomic data provided by the Human Protein Atlas (https://www.proteinatlas.org/ENSG00000145632-PLK2/single+cell/ovary#detail_marker). The UMAP clustering plot and expression heatmap revealed that PLK2 is most prominently expressed in endothelial cells (Cluster c-10), with levels significantly higher than in other cell types. This human-derived data provides reliable corroborative evidence for our findings from the mouse model, suggesting that PLK2 may serve as a conserved metabolic regulator across species (**Supplementary Figure S5**).

### 3.5 Transcriptome characterization and functional annotation of the PLK2 stratified PCOS subgroups

**Figure 4:**
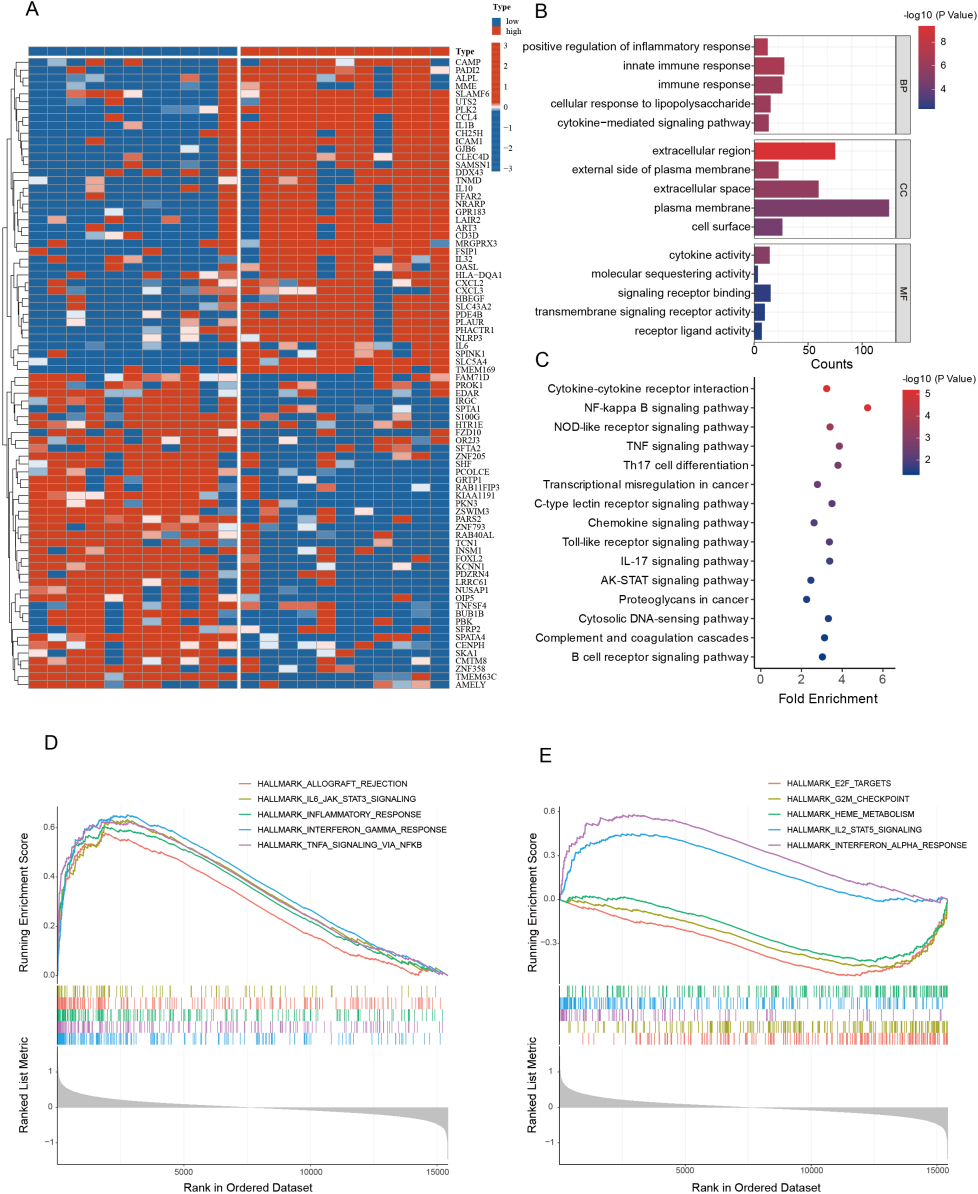
Functional enrichment analysis of DEGs between high and low PLK2 expression groups in PCOS. **(A)** The heatmap illustrates the expression profiles of DEGs between the high and low PLK2 expression groups in PCOS. Red represents high expression, while blue indicates low expression. **(B)** GO enrichment analysis of DEGs, highlighting significantly enriched biological processes (BP), cellular components (CC), and molecular functions (MF). The x-axis represents the number of enriched genes, and the color gradient indicates statistical significance (-log10 P-value). **(C)** KEGG pathway enrichment analysis of DEGs. The x-axis denotes fold enrichment, while the color gradient reflects statistical significance (-log10 P-value). **(D, E)** Hallmark gene set enrichment analysis illustrating the core biological characteristics associated with DEGs.

To delineate PLK2-associated molecular signatures, we stratified PCOS samples into high- and low-PLK2 subgroups. Volcano plot analysis identified 246 significantly upregulated and 150 downregulated genes (|log2FC| > 0.6, *P* < 0.05) in the high-PLK2 group. Heatmap visualization of the top 40 DEGs revealed that the high-PLK2 subgroup exhibited upregulation of pro-inflammatory mediators (IL1B, IL6, CXCL2/3), glycolytic enzymes (LDHA), immune cell recruitment factors (ICAM1), and ECM remodeling factors (PLAUR, PHACTR1). In contrast, the low-PLK2 subgroup was enriched for antifibrotic (SFRP2), hormone regulatory (FOXL2), cell cycle control (BUB1B), and glucose metabolism (SLC5A4) genes (**Figure 4A**).

GO enrichment analysis showed significant associations of high-PLK2 genes with inflammatory pathways (“positive regulation of inflammatory response”, enrichment ratio = 6.90, *P*=1.19 × 10^−7^), extracellular region (enrichment ratio = 2.02, P = 4.29 × 10^−10^), and cytokine activity (enrichment ratio = 4.30, *P*=5.13 × 10⁻⁶) (**Figure 4B**). KEGG pathway analysis highlighted activation of “NF-κB signaling” (enrichment ratio = 5.25, *P*=1.56 × 10⁻⁵) and “IL-17 signaling” (enrichment ratio = 3.38, *P*=0.0169), consistent with chronic inflammatory mechanisms (**Figure 4C**). Hallmark gene set enrichment analysis further demonstrated activation of pro-inflammatory pathways (TNF-α signaling: NES=2.79; interferon-γ response: NES=2.90; inflammatory response: NES=2.15), whereas cell cycle regulatory pathways were suppressed (G2M checkpoint: NES = -2.15; E2F targets: NES = -2.41) (**Figures 4D, E**). These findings suggest that PLK2 is central to PCOS pathogenesis by driving inflammation, fibrosis, and metabolic reprogramming.

### 3.6 Immune response profiling based on PLK2 expression in PCOS

Given the pivotal role of PLK2 in inflammatory fibrosis, we applied a combination of CIBERSORT, ESTIMATE, and ssGSEA algorithms to explore PLK2-related immune responses in PCOS. It is important to note that the bulk RNA-seq data analyzed here were derived from ovarian granulosa cells, which are not immune cells themselves but may exhibit transcriptional responses to immune stimuli, particularly under inflammatory conditions. Thus, the analyses performed reflect immune-related gene expression activity rather than true tissue-level immune cell infiltration.

**Figure 5:**
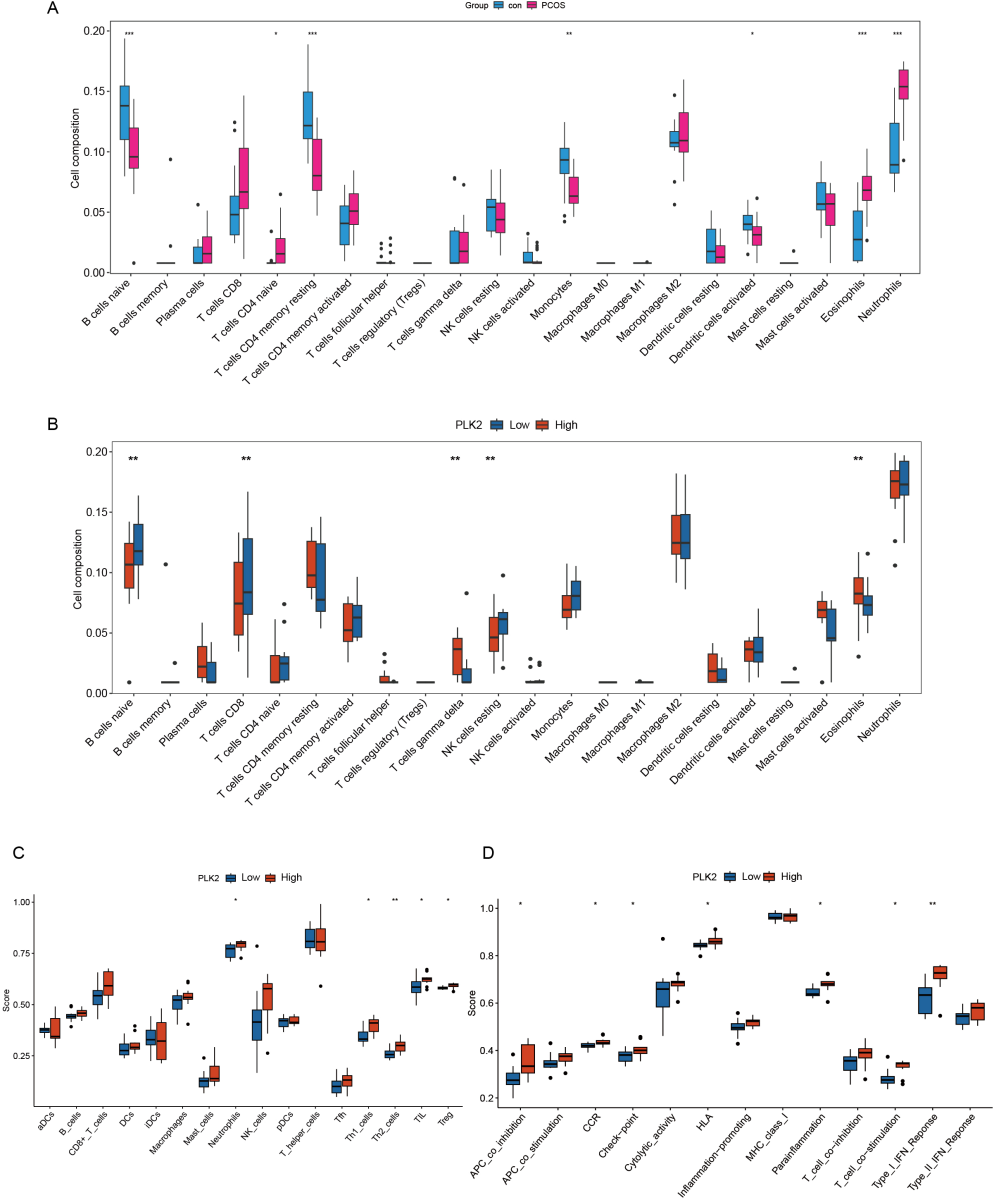
PLK2-associated immune-related transcriptomic activity in PCOS. **(A, B)** Immune cell-associated expression features inferred using the CIBERSORT algorithm. **(A)** Comparison of immune cell expression signals between PCOS and control groups. The y-axis represents the estimated proportion of immune cells, and the x-axis lists different immune cell types. **(B)** Comparison of immune cell-related expression differences between high and low PLK2 expression subgroups. Significant differences are indicated with corresponding statistical markers. **(C, D)** Enrichment analysis of immune functional pathways performed using the ssGSEA method. **(C)** Boxplots show immune cell scores between PLK2 expression subgroups. **(D)** Enrichment scores of immune functional pathways associated with PLK2 expression; the y-axis represents pathway activity scores, and statistical significance is indicated accordingly. Statistical significance is indicated as * *P* < 0.05, ** *P* < 0.01 and *** *P* < 0.001.

CIBERSORT analysis revealed a significant increase in neutrophils (*P* < 0.01) and a reduction in naive B cells, memory CD4+ T cells, and monocytes in PCOS (**Figure 5A**). Stratification based on PLK2 expression further demonstrated elevated eosinophil and γδ T-cell infiltration in the high-PLK2 subgroup (*P* < 0.01), along with decreased naive B cells and CD8+ T cells, indicating immune homeostasis imbalance (**Figure 5B**).

Functional annotation via ssGSEA highlighted immune dysregulation in the high-PLK2 subgroup, characterized by a persistent inflammatory microenvironment. Notably, neutrophil abundance was significantly increased (*P* < 0.01), accompanied by concurrent elevations in Th1 and Th2 cells (*P* < 0.05) and activation of T-cell co-stimulation and type I interferon responses (*P* < 0.05) (**Figures 5C, D**).

### 3.7 Validation of PLK2 in a DHEA-induced PCOS rat model

**Figure 6:**
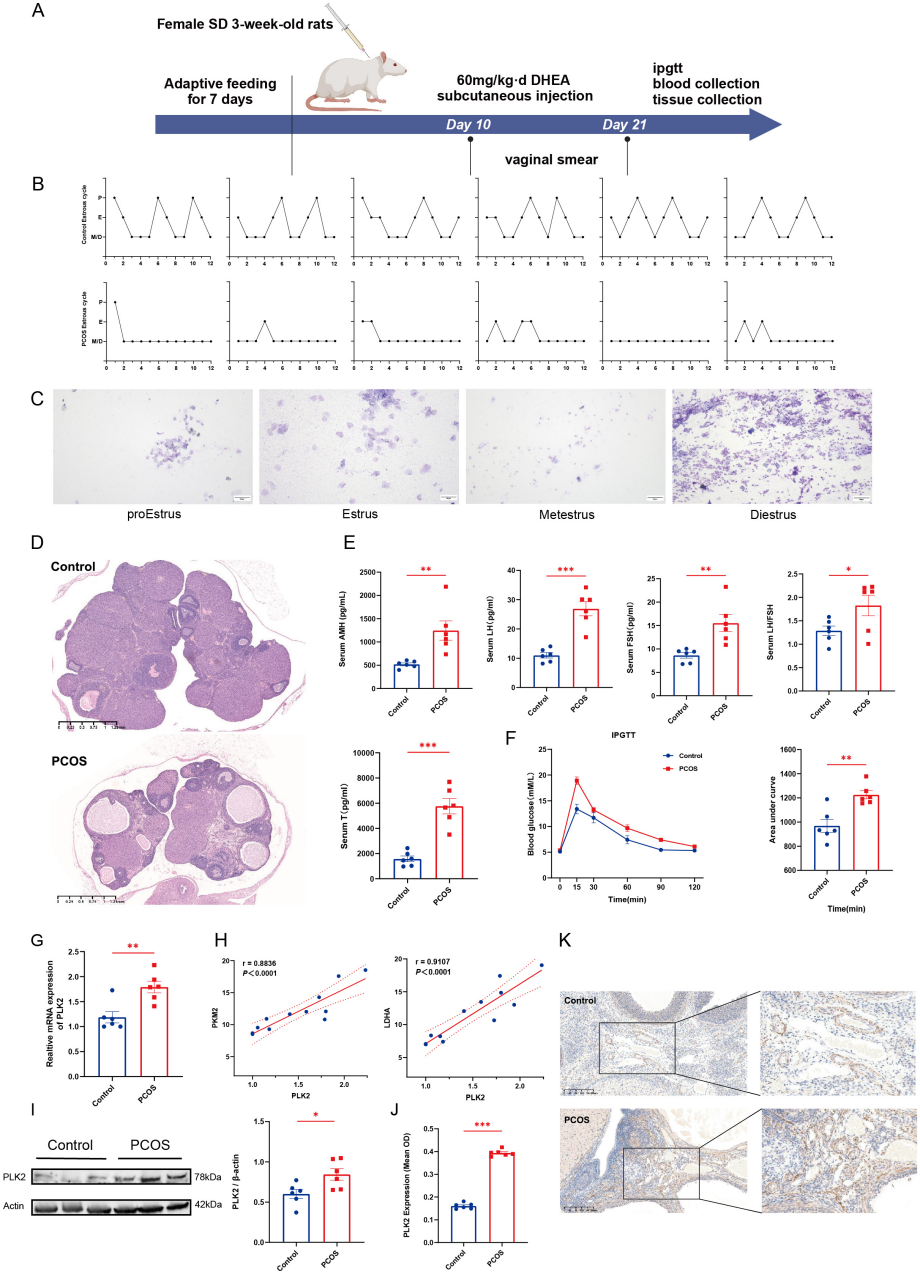
Validation of PLK2 in the rat PCOS model. **(A)** Schematic of DHEA-induced PCOS rat model (Made using ^©^BioRender-biorender.com). **(B)** Motility cycle monitoring in control and PCOS rats. **(C)** Representative images of vaginal smears depicting the four phases of the estrous cycle: pre-estrus, estrus, post-estrus, and menopause. **(D)** H&E staining of ovarian sections from control and PCOS rats. **(E)** Serum hormone levels of control and PCOS rats, including AMH, LH, FSH, and T levels. **(F)** IPGTT results and quantitative analysis of the area under the curve. **(G)** Relative mRNA expression levels of PLK2 in ovarian tissues. **(H)** Correlation analysis between PLK2 expression and key glycolytic enzymes (LDHA and PKM2). **(I)** Protein levels of PLK2 in ovarian tissues. **(J)** Quantification of average optical density (OD) of PLK2 protein expression in ovarian endothelial tissues. **(K)** Immunohistochemical staining of PLK2 in ovarian endothelial cells * *P* < 0.05, ** *P* < 0.01 and *** *P* < 0.001.

To validate the critical role of PLK2 in PCOS pathogenesis, we established a DHEA-induced hyperandrogenic rat model (n = 6) to recapitulate androgen excess and ovarian dysfunction characteristic of PCOS (**Figure 6A**). This model consistently exhibited core PCOS phenotypes, including disrupted estrous cycles (prolonged diestrus phase, *P* < 0.01) (**Figures 6B, C**) and polycystic ovarian morphology, characterized by cystic follicles and thickened theca layers (**Figure 6D**). Hormonal profiling indicated significantly elevated serum T levels (*P* < 0.0001), AMH, and LH/FSH ratio (*P* < 0.01), suggesting hyperandrogenemia and ovulatory dysfunction (**Figure 6E**). Additionally, DHEA-treated rats displayed impaired glucose metabolism, as evidenced by increased AUC in IPGTT (**Figure 6F**).

PLK2 was significantly upregulated in the ovaries of DHEA-exposed PCOS rats. Quantitative PCR demonstrated a marked increase in PLK2 mRNA expression compared to the control group (*P*=0.0038) (**Figure 6G**). The Western blot results showed that PLK2 protein levels were significantly elevated in PCOS ovaries compared to the control group (**Figure 6I**). Immunohistochemical analysis further localized PLK2 protein overexpression to endothelial cells within the ovarian stroma (**Figures 6J, K**, P < 0.0001). Correlation analysis between PLK2 and ovarian glycolysis was performed to elucidate further the relationship between PLK2 and the key glycolytic enzyme LDHA (**Figure 6H**, r = 0.9107, *P* < 0.0001). These findings establish PLK2 as a pivotal regulator of aberrant glycolysis in PCOS, playing a crucial role in disease progression.

### 3.8 Knockdown of PLK2 significantly attenuates DHEA-induced upregulation of glycolytic genes

**Figure 7:**
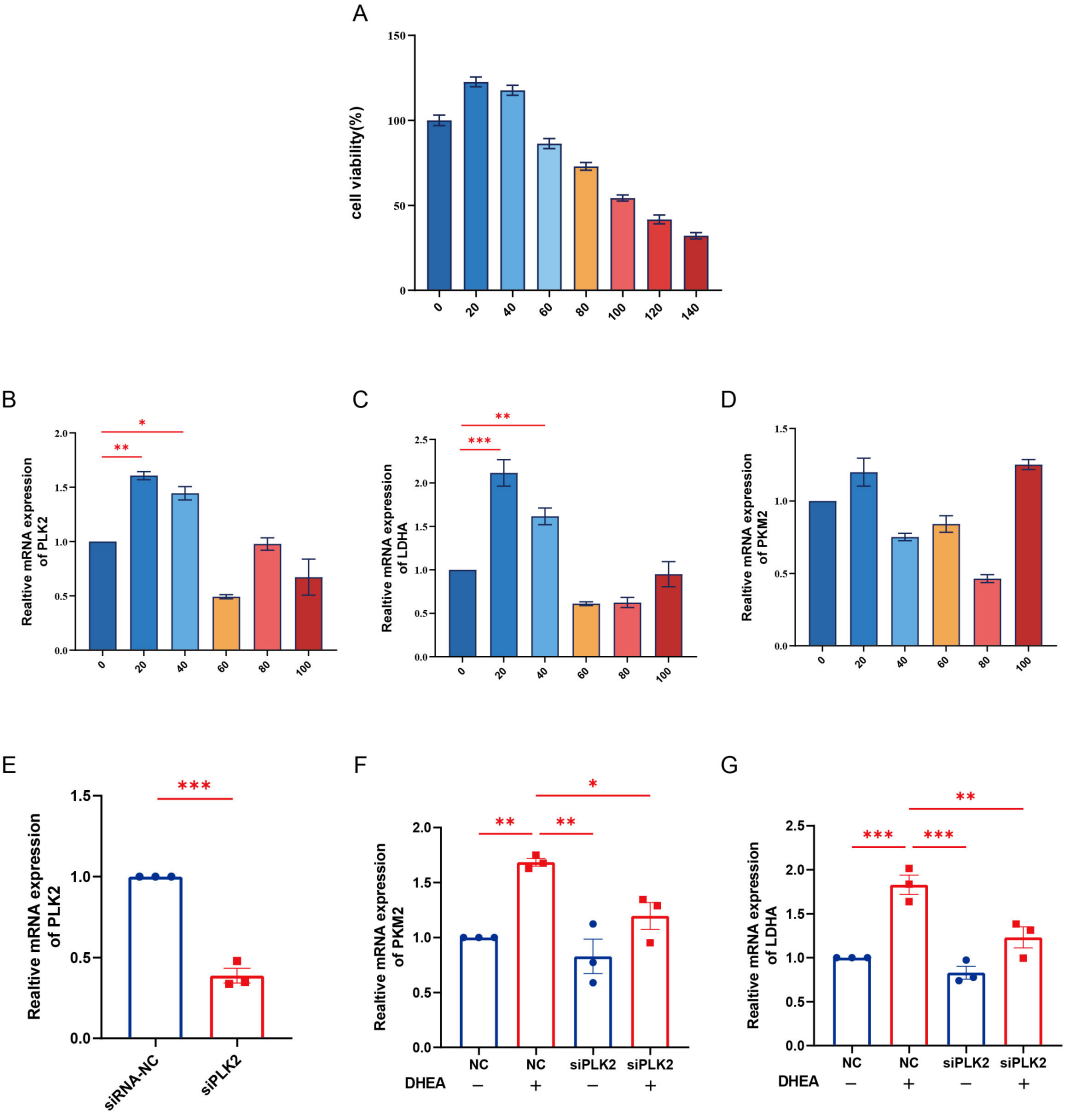
Knockdown of PLK2 attenuates DHEA-induced upregulation of glycolytic genes in HUVECs. **(A)** Cell viability of HUVECs treated with different concentrations of DHEA (0-140 μM) for 24 h was assessed by the CCK-8 assay. **(B–D)** mRNA expression levels of PLK2, LDHA, and PKM2 in HUVECs after treatment with various concentrations of DHEA, as determined by qPCR. **(E)** Verification of PLK2 knockdown efficiency by qPCR in HUVECs transfected with siPLK2 or siRNA-NC. **(F–G)** Effects of PLK2 knockdown on the mRNA expression of LDHA and PKM2 in the presence or absence of DHEA (20 μM). * *P* < 0.05, ** *P* < 0.01 and ***P < 0.001.

To mimic the hyperandrogenic environment associated with PCOS, HUVECs were treated with different concentrations of DHEA, and cell viability was assessed using the CCK-8 assay. The results showed that 20 μM DHEA markedly increased the expression of PLK2 and key glycolytic enzymes (LDHA, PKM2) without significantly affecting cell proliferation, and this concentration was therefore selected for subsequent experiments (**Figures 7A–D**). Knockdown of PLK2 via siRNA transfection was confirmed by qPCR (**Figure 7E**), showing more than 60% reduction in PLK2 mRNA levels (*P* < 0.01). Functional analysis demonstrated that PLK2 silencing partially reversed the DHEA-induced upregulation of LDHA and PKM2 (**Figures 7F, G**, P < 0.05). These findings indicate that PLK2 participates in the regulation of glycolytic enzyme expression under hyperandrogenic conditions and may play an important role in PCOS-related metabolic dysregulation.

## 4 Discussion

Glycolysis is pivotal in cellular metabolism and has been closely linked to PCOS pathophysiology (14, 26). Through single-cell RNA sequencing, we identified endothelial cells as the key glycolytic population within the ovarian stroma of PCOS patients. Cell-cell communication analysis revealed that endothelial cells are central hubs in the dysregulated intercellular network, fostering inflammation and extracellular matrix (ECM) remodeling. Transcriptomic analysis identified PLK2 as a key regulator of glycolytic imbalance in PCOS endothelial cells, with its high expression correlating with immune dysregulation. Animal studies further validated PLK2 upregulation in PCOS models and its association with glycolytic enzymes markers. Furthermore, *in vitro* functional assays demonstrated the indispensable role of PLK2 in mediating glycolysis in endothelial cells under hyperandrogenic exposure, providing strong evidence that PLK2 acts as a crucial regulator of PCOS-associated metabolic disturbances.

Endothelial cells primarily rely on glycolysis to generate ATP, supporting proliferation, migration, and angiogenesis (27). Dysregulation of glycolytic enzymes profoundly affects endothelial function. For instance, PFKFB3 induces endothelial-to-mesenchymal transition (EndMT) by disrupting mitochondrial respiration, contributing to tissue fibrosis (28). Enhanced endothelial glycolysis also promotes inflammation, a feature consistent with the extensive vascular network in ovarian tissue that facilitates immune cell trafficking. Endothelial dysfunction in PCOS has been largely studied in the context of cardiovascular complications, where it is considered an early marker of vascular disease (29). Keller et al. (30). first identified endothelial dysfunction in a DHT-induced PCOS rat model, marked by increased vasoconstrictive prostaglandin activity and impaired vasodilation. Victor et al. (31). further demonstrated that insulin resistance exacerbates oxidative stress, enhancing leukocyte-endothelial interactions and promoting endothelial dysfunction in PCOS patients. Similarly, Zuo et al. (32). reported endothelial dysfunction and ovarian fibrosis in single-cell transcriptomic analyses. Our study expands upon these findings by highlighting the central role of endothelial glycolytic dysregulation, linking endothelial dysfunction with metabolic and immune abnormalities in PCOS.

Cell-cell communication analysis revealed intricate interactions between endothelial cells and other ovarian cell types, positioning endothelial cells as key regulators of immune responses and tissue remodeling in PCOS. Aberrant endothelial-fibroblast interactions have been implicated in inflammation and fibrosis-related diseases, with endothelial cells secreting cytokines such as IL-6 and CCL2 or engaging in ligand-receptor interactions to modulate immune cell infiltration (33). Glycolysis-driven endothelial dysfunction and altered intercellular communication have also been observed in cardiovascular and neurological disorders. Lipopolysaccharide-induced inflammation enhances glycolytic activity and increases its metabolic byproduct lactate in neutrophils (34). Endothelial-derived lactate promotes the transformation of macrophages into a pro-angiogenic and pro-regenerative M2-like phenotype (35, 36). In PCOS, heightened endothelial-fibroblast and endothelial-immune interactions likely contribute to chronic inflammation and immune dysregulation, forming a pathological feedback loop that exacerbates ovarian dysfunction. Targeting endothelial cell communication pathways may provide novel therapeutic strategies for restoring immune balance and metabolic homeostasis in PCOS.

PLK2 is a highly conserved serine/threonine kinase predominantly studied in neurodegenerative diseases and cancer, with limited research on its role in glycolysis and inflammation. PLK2 is an early response gene in microglia following LPS stimulation, and its knockdown significantly attenuates LPS-induced pro-inflammatory cytokine expression (37). Additionally, PLK2 promotes renal fibrosis in diabetic nephropathy via Notch signaling activation (38). However, its role in PCOS remains largely unexplored. Our findings suggest that PLK2 may exert similar regulatory effects on inflammation and fibrosis within ovarian glycolytic dysfunction, particularly in endothelial cells.

Clustering analysis indicated that PLK2 plays a key role in immune regulation, with its high-expression subgroup significantly enriched in pro-inflammatory pathways involved in immune cell recruitment and activation. These results align with studies demonstrating PLK2-mediated inflammation in pulmonary fibrosis (39) and glioblastoma (40). Beyond immune dysregulation, PLK2 is an emerging regulator of fibroblast function and fibrosis (39). Transcriptomic GO and KEGG analyses revealed strong associations between PLK2 expression and ECM remodeling pathways. In high-PLK2 subgroups, collagen-related ECM components and PLAUR were significantly upregulated, promoting ECM deposition and fibrosis, thereby impairing follicular development and ovarian function. Moreover, elevated NF-κB and IL-17 signaling in high-PLK2 patients further supports its role in inflammation-driven fibrosis.

Our immune-related transcriptomic analysis based on granulosa cell data revealed substantial heterogeneity in immune signaling activity among PCOS patients, particularly pronounced in the PLK2-high subgroup. These findings corroborate Deng et al. (41). report, reinforcing the concept that chronic low-grade inflammation not only represents a core pathological feature of PCOS but also exacerbates metabolic and reproductive dysfunction. The high-PLK2 subgroup exhibited significantly increased neutrophil infiltration, consistent with prior studies implicating neutrophil-driven inflammation in oxidative stress and ovarian dysfunction (42). Additionally, elevated Th1 and type I interferon responses further confirmed PLK2’s role in shaping the pro-inflammatory microenvironment, potentially contributing to follicular atresia and ovulatory dysfunction (43). Notably, the high-PLK2 subgroup significantly reduced CD8+ T cells and naive B cells, suggesting impaired immune surveillance, which may perpetuate chronic inflammation and pathological ovarian remodeling (44). Furthermore, upregulation of ICAM1 and CXCL2 in PLK2-high patients likely facilitates excessive immune cell recruitment, exacerbating ovarian inflammation. These findings align with the hypothesis that immune dysregulation drives PCOS progression, reinforcing the intricate interplay between metabolic and inflammatory perturbations (45).

The interplay between endothelial dysfunction, glycolytic reprogramming, immune imbalance, and PLK2 provides a comprehensive perspective on PCOS pathogenesis. As emphasized by Rudnicka et al. (46), chronic low-grade inflammation exacerbates endothelial dysfunction, which disrupts glycolysis. Our study extends this concept by proposing PLK2 as a key mediator linking endothelial glycolytic dysregulation and immune imbalance in PCOS.

Previous studies have demonstrated that glycolytic activation in endothelial cells plays a pivotal role in fibrosis and inflammation. For example, PFKFB3-driven glycolysis has been shown to trigger endothelial-to-mesenchymal transition, leading to fibrotic responses, while pharmacological inhibition effectively suppresses this process (28). Additionally, endothelial glycolysis enhances the production of pro-inflammatory cytokines and growth factors (47), and contributes to inflammatory angiogenesis (48). These findings strongly corroborate our hypothesis that PLK2 may link endothelial metabolic imbalance with inflammation and extracellular matrix remodeling.

*In vivo*, we observed a significant upregulation of PLK2 expression in the ovaries of PCOS model rats, positively correlated with key glycolytic enzymes. This suggests that under hyperandrogenic conditions, PLK2 not only reflects glycolytic activation but may also regulate inflammatory responses and tissue remodeling via metabolic pathways, thereby disrupting follicle development and ovulation. *In vitro*, PLK2 silencing markedly attenuated DHEA-induced upregulation of glycolytic enzymes, reinforcing its central role in maintaining glycolytic activation under androgenic exposure, consistent with the *in vivo* findings. Taken together, these observations suggest that PLK2 is not merely a pathological marker but may function as a key regulator coupling glycolysis and inflammation. We propose that PLK2 might partake in a glycolysis–inflammation–ECM remodeling positive feedback loop, exacerbating local metabolic and immune dysregulation in the ovary. Targeting PLK2 may thus restore glycolytic homeostasis and present a novel therapeutic strategy for addressing chronic inflammation and tissue remodeling in PCOS.

Several therapeutic strategies targeting glycolysis have shown promise in PCOS management. For instance, dendrobium polysaccharides ameliorate insulin resistance in granulosa cells by activating glycolysis, thereby improving ovulatory dysfunction (49). Similarly, nicotinamide mononucleotide (NMN) modulates glycolysis via the PI3K/AKT pathway, alleviating insulin resistance and restoring endometrial homeostasis (26). Additionally, a combination of Diane-35 and metformin enhances ovarian energy metabolism by regulating glycolytic pathways (17). Our findings further emphasize the potential of targeting endothelial glycolytic dysregulation as a novel therapeutic approach for PCOS treatment.

## 5 Conclusion

Our study systematically elucidates the pivotal role of endothelial cells in the glycolytic reprogramming associated with PCOS, and identifies PLK2 as a key regulatory factor linking endothelial dysfunction, enhanced glycolysis, and immune imbalance. These findings provide novel insights into the pathophysiological mechanisms of PCOS. To further validate and expand upon these findings, future studies should incorporate stromal cell-specific transcriptomic datasets, particularly human single-cell RNA sequencing data, to gain a more comprehensive understanding of stromal cell involvement in PCOS. In addition, the precise molecular mechanisms by which PLK2 regulates glycolysis in endothelial cells remain to be fully elucidated. Targeted exploration of PLK2-related signaling pathways may offer promising therapeutic avenues to mitigate glycolytic dysregulation, chronic inflammation, and ovarian dysfunction in PCOS.

Despite the strengths of this study, several limitations should be acknowledged. First, the single-cell RNA sequencing dataset (GSE268919) used in this work was derived from a DHEA-induced mouse PCOS model. Although this model has been well characterized and is widely applied, interspecies differences may limit its ability to fully recapitulate the transcriptional features of the human ovarian stroma. Therefore, future studies should integrate single-cell datasets from human ovarian stromal cells to enhance the clinical translational relevance of the findings. Second, the relatively small sample size in the single-cell analysis may affect statistical robustness and the generalizability of the results. Additionally, the bulk RNA-seq data used for immune and metabolic analyses were derived from granulosa cells. While this provides valuable insights into PCOS-associated molecular alterations, granulosa cells are not stromal cells, and thus cannot fully reflect stroma-specific expression patterns. Currently, publicly available human ovarian stroma datasets remain limited. No interventional experiments targeting PLK2 were performed, limiting the ability to definitively determine its regulatory role. Lastly, although PLK2 is implicated in PCOS-associated endothelial metabolic abnormalities and inflammatory activation, its downstream regulatory pathways remain to be elucidated. Future work will employ shRNA-mediated knockdown and mechanistic studies to further clarify the critical role of PLK2 in ovarian stromal glycolysis and the pathogenesis of PCOS.

## Data Availability

The original contributions presented in the study are included in the article/**Supplementary Material**. Further inquiries can be directed to the corresponding author.
