# Supplementary material for: PLK2 as a key regulator of glycolysis and immune dysregulation in polycystic ovary syndrome
Source: Front Immunol. 2025 Sep 11;16:1610713. doi: 10.3389/fimmu.2025.1610713 (PMC12460256; doi:10.3389/fimmu.2025.1610713)
Supplement: Supplementary file 1 [file DataSheet1.docx]

Supplementary Material

# Supplementary Tables

**Supplementary Table 1. Transfected siRNA in this study.**

|  | Forward | Reverse |
| --- | --- | --- |
| NC | UUCUCCGAACGUGUCACGUTT | ACGUGACACGUUCGGAGAATT |
| PLK2-Homo-1093 | CCAGUUUGGAUGACAUCAUTT | AUGAUGUCAUCCAAACUGGTT |

**Supplementary Table 2. qRT-PCR primers in this study.**

| Gene | Species | Forward | Reverse |
| --- | --- | --- | --- |
| PLK2 | Rat | ACAGTGGCAAGAGTCCTTCG | GAGCTGGTATCCAAAGCCGT |
| LDHA | Rat | GCAATCTGGATTCGGCTCGGTTC | CGGCGACATTCACACCACTCC |
| PKM2 | Rat | GTGCCGCCTGGACATTGACTC | ATTCAGCCGAGCCACATTCATCC |
| β-actin | Rat | CTAAGGCCAACCGTGAAAAG | ACCAGAGGCATACAGGGACA |
| PLK2 | Human | CCGTCGGTGTCCTTTTCAACA | CTCCACCATCCATGAGGTTCT |
| LDHA | Human | ATGGCAACTCTAAAGGATCAGC | CCAACCCCAACAACTGTAATCT |
| PKM2 | Human | ACTGGCATCATCTGTACCATTG | AGCCACATTCATTCCAGACTTA |
| β-actin | Human | GCGGGAAATCGTGCGTGAC | CAGGAAGGAAGGCTGGAAGAGTG |

#
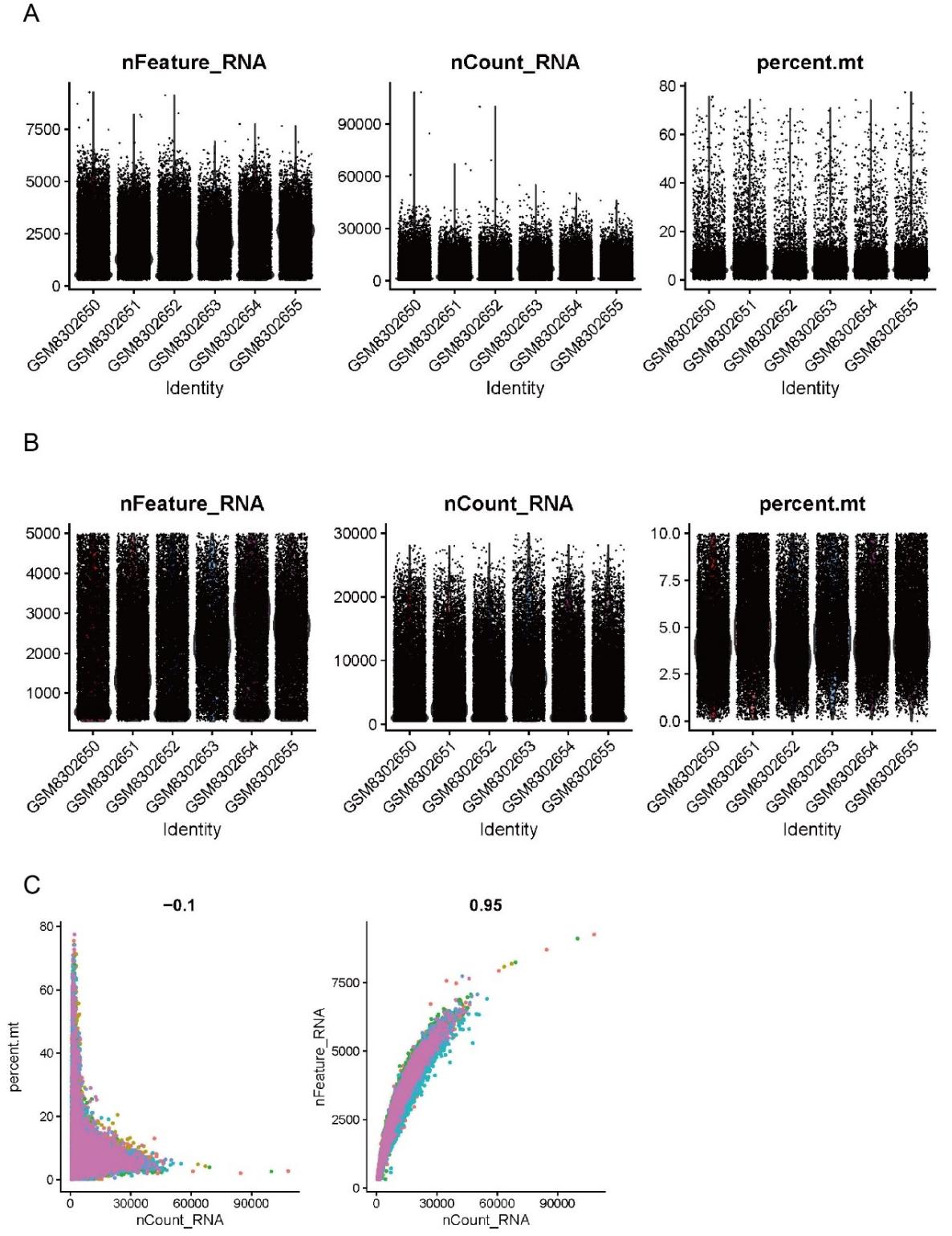
Supplementary Figures

**Supplementary Figure 1.** Quality control of single-cell RNA-seq data. (A) Violin plots showing distributions of nFeature_RNA, nCount_RNA, and percent.mt across samples before filtering. (B) Violin plots showing distributions of nFeature_RNA, nCount_RNA, and percent.mt after filtering. (C) Scatter plots illustrating correlations among quality control metrics


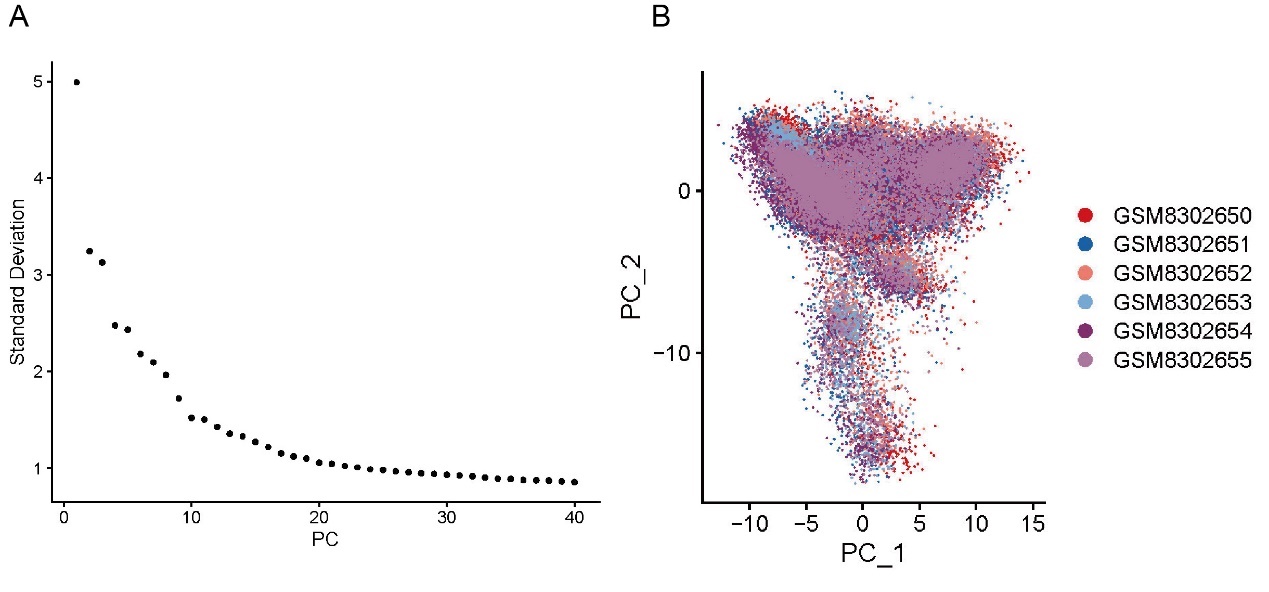


**Supplementary Figure 2.** PCA of scRNA-seq data. (A) Elbow plot showing the standard deviation of principal components. (B) PCA plot of cells based on the first two principal components, colored by sample.


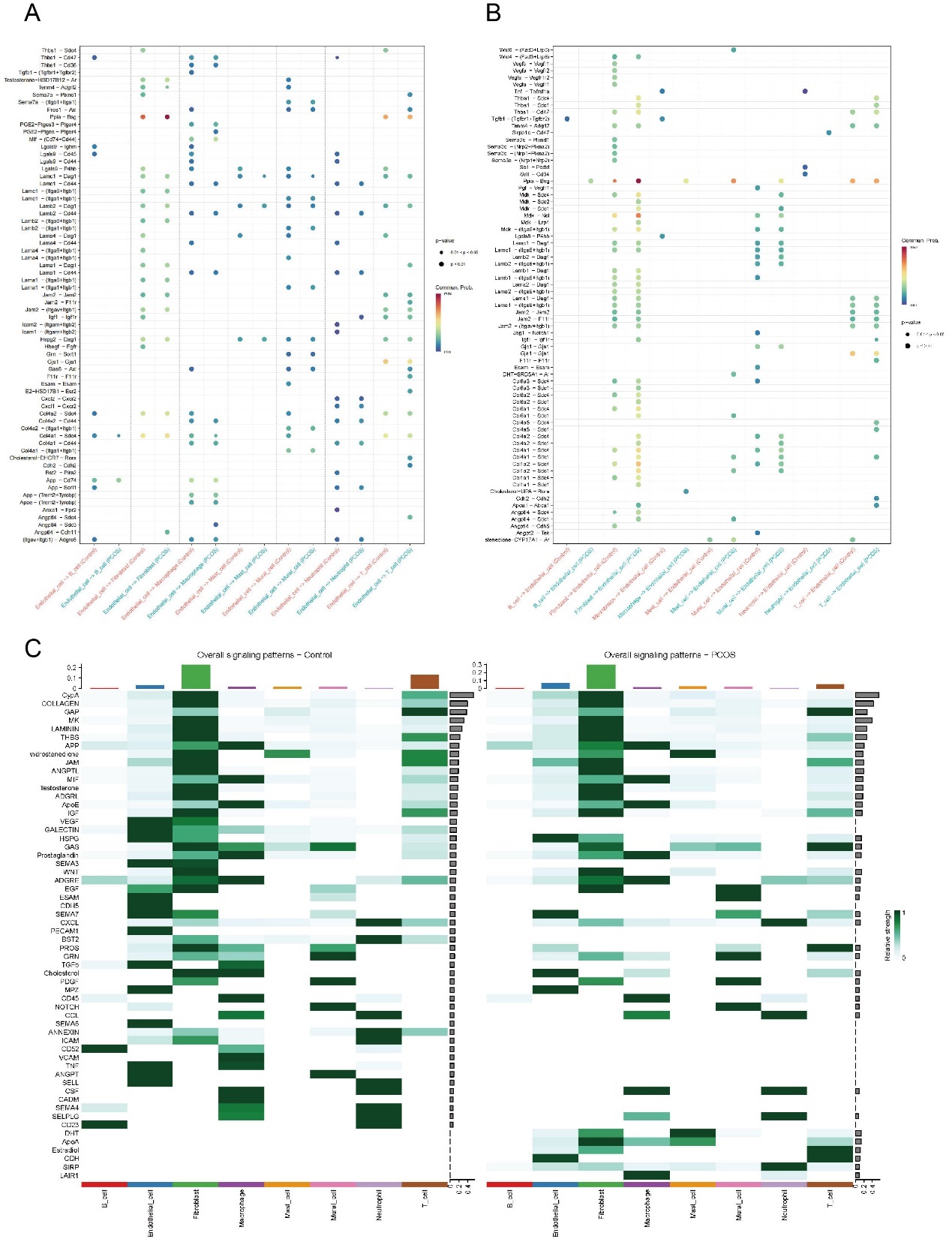
**Supplementary Figure 3.** Inferred cell-cell communication networks from scRNA-seq. (A) Dot plot of ligand–receptor interactions across cell types in the control group. (B) Dot plot of ligand–receptor interactions across cell types in the PCOS group. (C) Heatmaps summarizing overall signaling strength between cell types in control and PCOS groups.


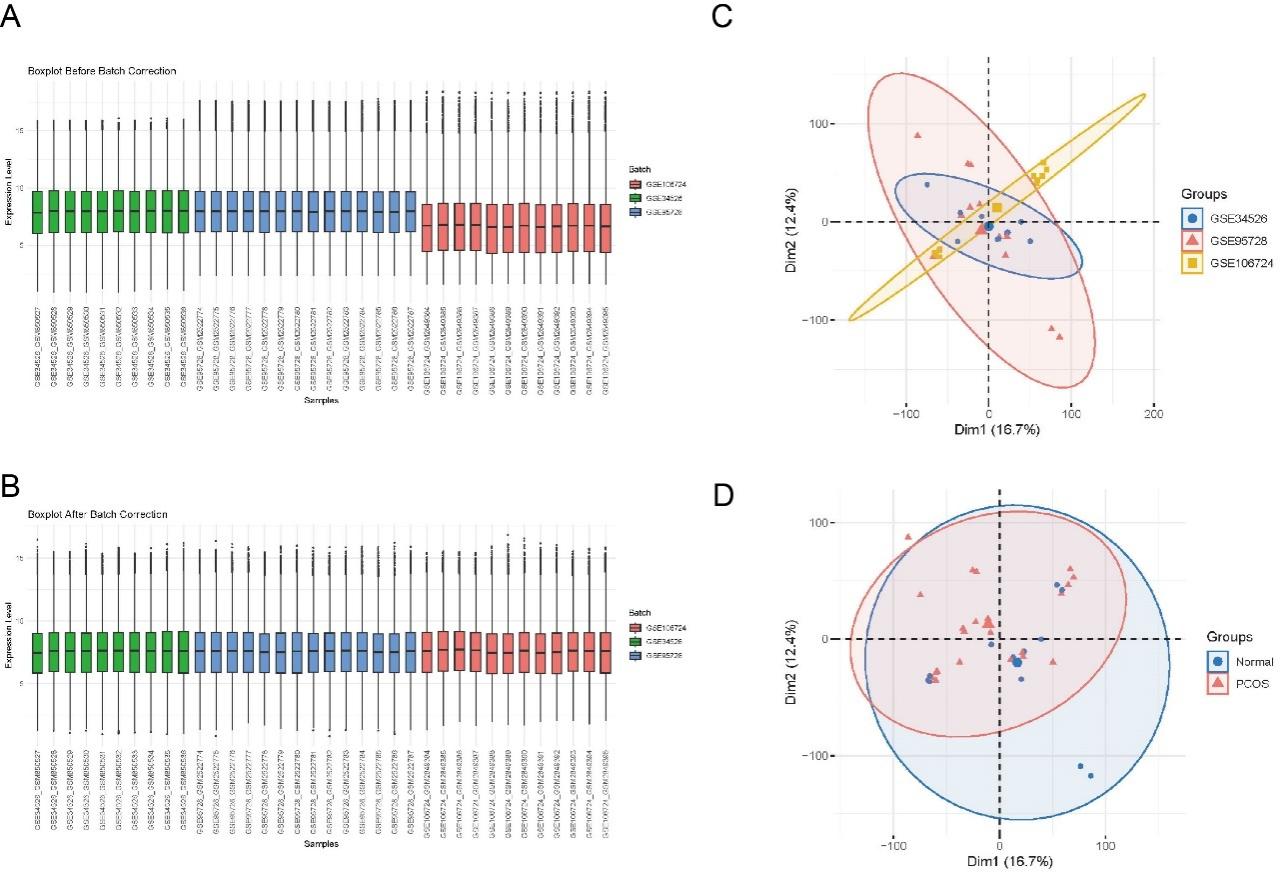


**Supplementary Figure 4.**Batch effect evaluation and correction of transcriptomic data. (A) Boxplots of gene expression across samples before batch correction. (B) Boxplots of gene expression across samples after batch correction. (C) PCA plot of samples before batch correction. (D) PCA plot of samples after batch correction.

**Supplementary Figure 5.** PLK2 expression profile across human ovarian cell types based on single-cell transcriptomic data from the Human Protein Atlas (HPA).

**
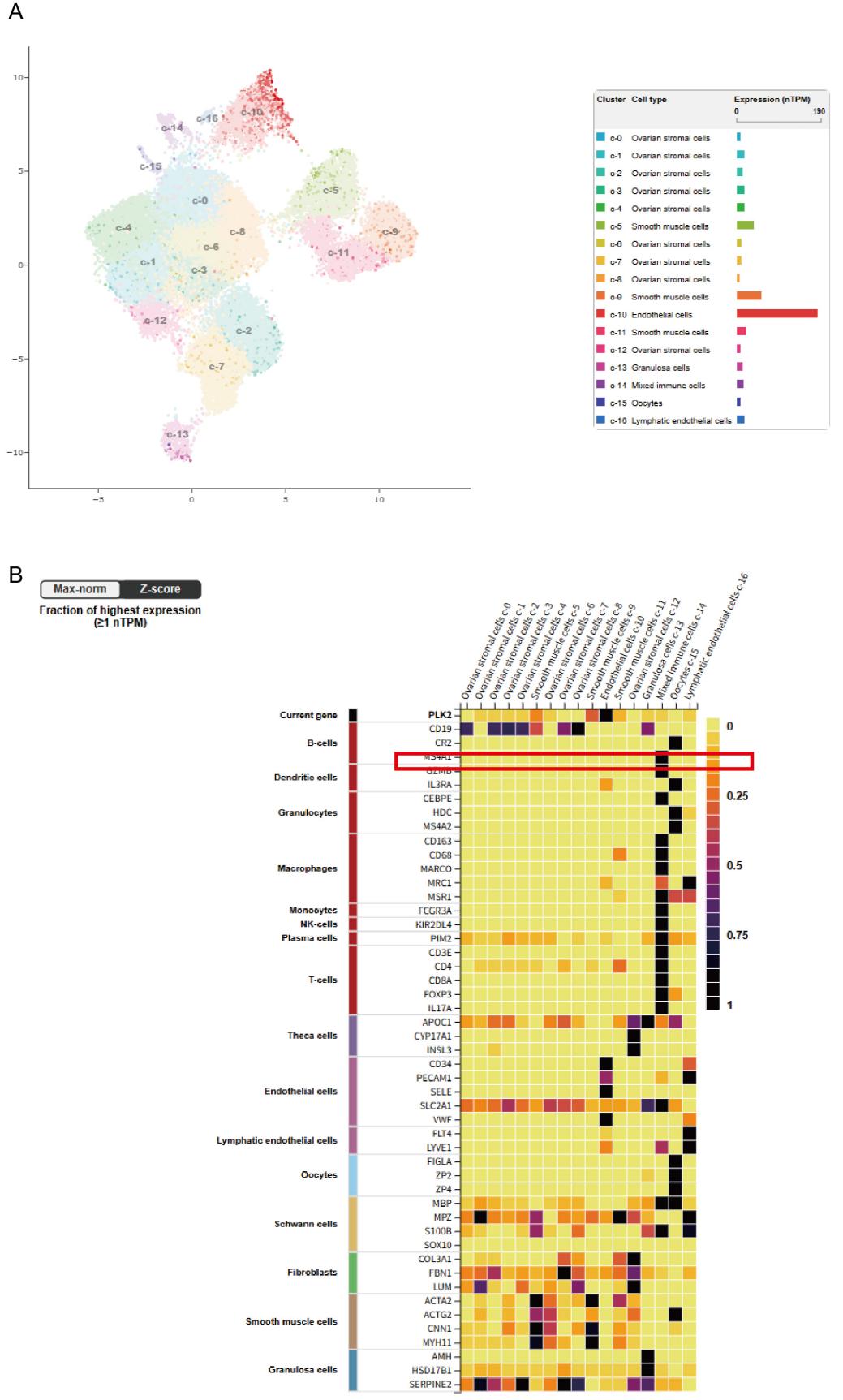
**(A) UMAP plot showing the clustering of ovarian cell populations (n=17 clusters) in human ovarian tissue. Cluster c-10 is annotated as endothelial cells and exhibits the highest PLK2 expression across all clusters.(B) Heatmap displaying normalized PLK2 expression across various human ovarian cell types.


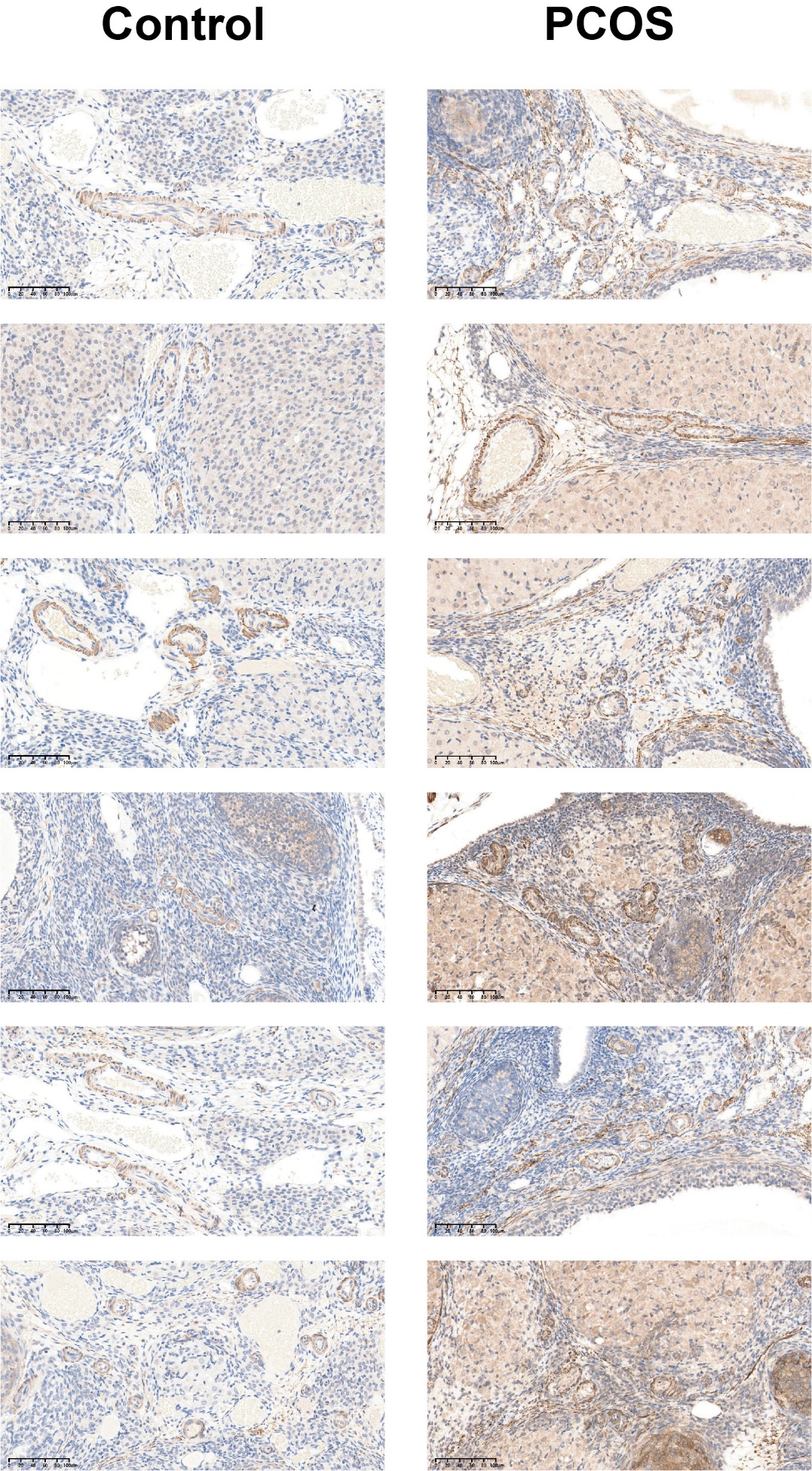


**Supplementary Figure 6.**. PLK2 expression in ovarian tissues of control and PCOS model rat.

Immunohistochemical staining of PLK2 in ovarian tissues from control and PCOS mice (n = 6 per group).
